# Selective laser melted titanium implants: a new technique for the reconstruction of extensive zygomatic complex defects

**DOI:** 10.1186/s40902-015-0001-9

**Published:** 2015-01-29

**Authors:** Horatiu Rotaru, Ralf Schumacher, Seong-Gon Kim, Cristian Dinu

**Affiliations:** 1 Department of Oral and Cranio-Maxillofacial Surgery, “Iuliu Hatieganu” University of Medicine and Pharmacy, Str. Motilor Nr. 33, 400001 Cluj-Napoca, Romania; 2 School of Life Sciences, Institute for Medical and Analytical Technologies, University of Applied Sciences and Arts Northwestern Switzerland, Muttenz, Switzerland; 3 Department of Oral and Maxillofacial Surgery, Gangneung-Wonju National University, Gangneung, South Korea

**Keywords:** Selective laser melting, Custom-made, Titanium implant, Zygoma reconstruction

## Abstract

The restoration of extensive zygomatic complex defects is a surgical challenge owing to the difficulty of accurately restoring the normal anatomy, symmetry, proper facial projection and facial width. In the present study, an extensive post-traumatic zygomatic bone defect was reconstructed using a custom-made implant that was made with a selective laser melting (SLM) technique. The computer-designed implant had the proper geometry and fit perfectly into the defect without requiring any intraoperative adjustments. A one-year follow-up revealed a stable outcome with no complications.

## Background

Craniofacial trauma, tumor resection and congenital deformities can result in zygomatic bone deficiencies. The reconstruction of the zygomatic bone is essential for the restoration of function and esthetics. The reduction of psychosocial morbidity is also an important issue [1]. Accurate restoration of the normal anatomy, symmetry, proper facial projection and facial width are the key points in orbito-zygomatic reconstruction [1].

Different surgical approaches had been described for the reconstruction of the zygomatic complex. These approaches include osteotomy, autologous bone graft, free tissue transfer and the use of different alloplastic implants [2]. Autologous bone grafts are still considered the gold standard for the reconstruction of these defects [3]. However, donor site morbidity, limited bone availability, unpredictable resorption rates, and residual deformities remain important challenges [4]. Different types of alloplastic implants, such as metals [5], silicone [6], polymers [7], and hydroxyapatite-based products [8], have been used to replace autologous bone grafts. However, the ideal alloplastic material has not yet been identified [7].

Although stock-made implants are commercially available in different sizes, these implants are of limited value for repairing acquired and unusual bony defects. Such implants fail to accurately fit the defects and hence result in outcomes that are associated with high revision rates [2,7]. In contrast, custom-made patient-specific implants that are produced using computer-aided design and manufacturing (CAD/CAM) overcome these drawbacks [7]. Patient-specific implants shorten the operative time, reduce the need for intraoperative implant adjustments and improve the clinical outcomes [9].

In this article, we present a case of post-traumatic zygomatic deficiency that has been successfully treated using a custom-made implant that was made with a selective laser melting (SLM) technique. After one year of follow-up, the implant exhibited good integration with no signs of infection or exposure. To the best of our knowledge, this case report is the first to describe a zygomatic reconstruction utilizing a custom-made implant that was created with the SLM technique.

## Case presentation

**Figure 1:**
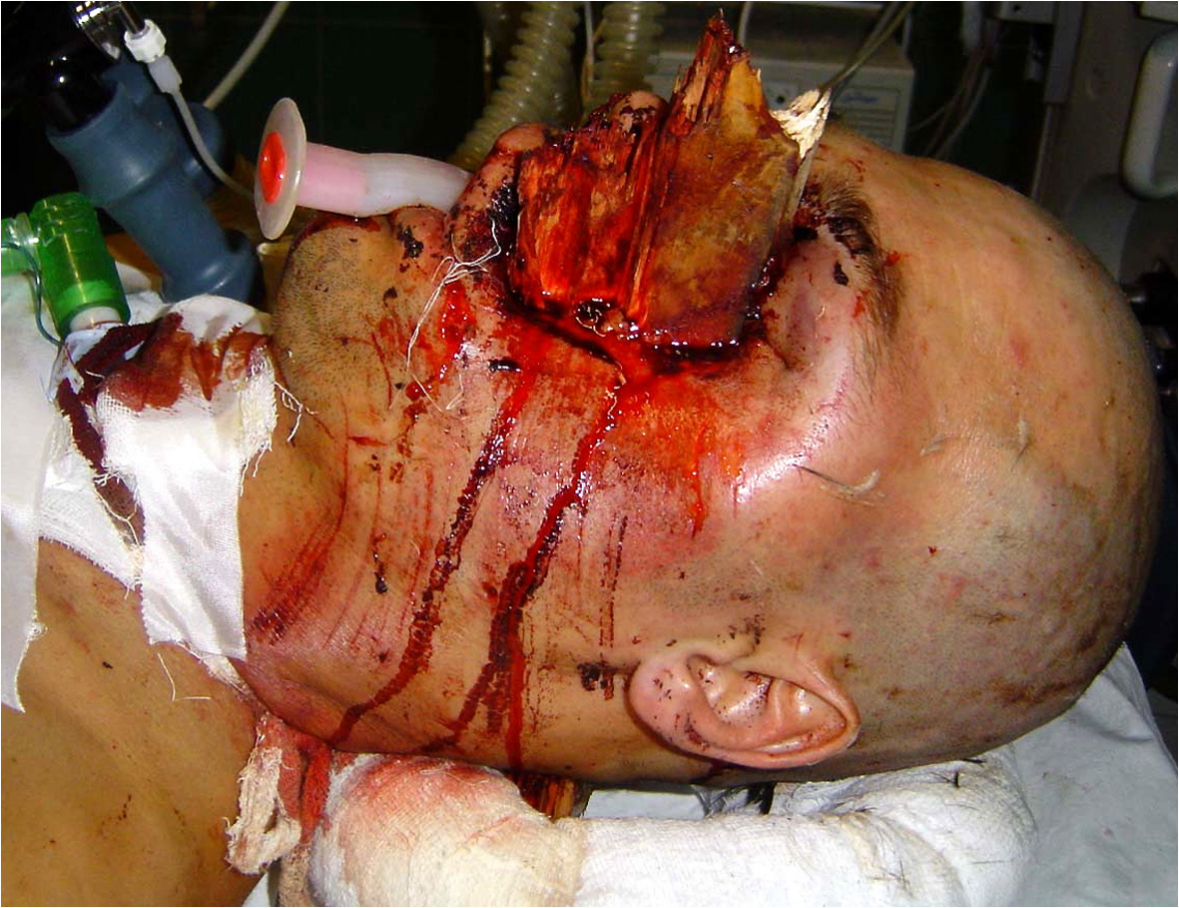
**Initial trauma event during which the bone segments were lost.**

**Figure 2:**
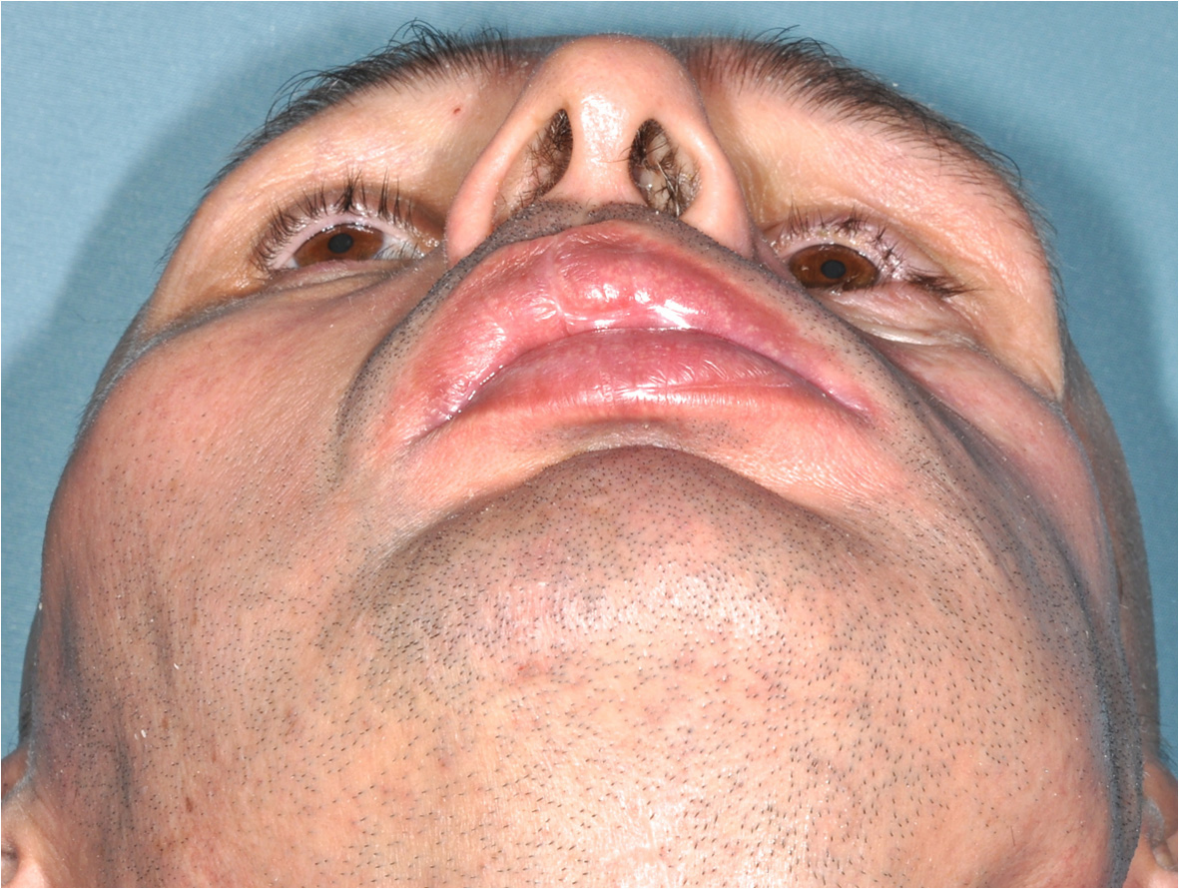
**Clinical appearance of the patient prior to implantation.** A depression of the left zygoma is noticeable.

**Figure 3:**
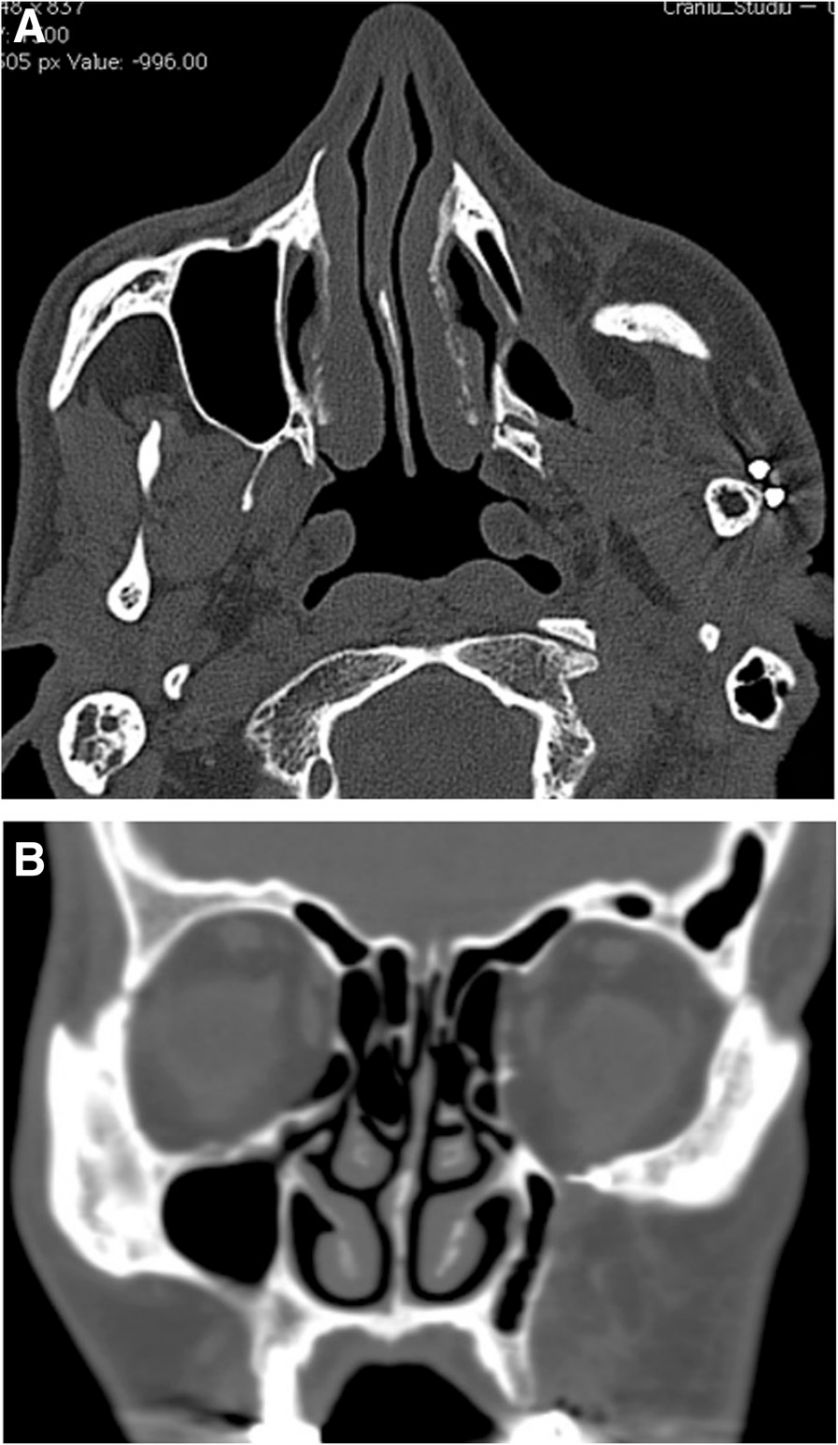
**CT scan confirming the left zygomatic deficiency: (A) Axial, (B) Coronal.**

A 43-year-old male patient presented to our department with a severe left midfacial post-traumatic deformity due to road traffic accident that occurred 6 years prior (Figure 1). Clinical examination of the left midface revealed the loss of the antero-posterior and medio-lateral (transverse) projections of the left zygomatic bone. A slight enophthalmos was also present. The soft tissues of the area were hypotrophic in response to the initial injury (Figure 2). The clinical findings were confirmed on computerized tomogram (CT) images in axial and coronal plane (Figure 3).

**Figure 4:**
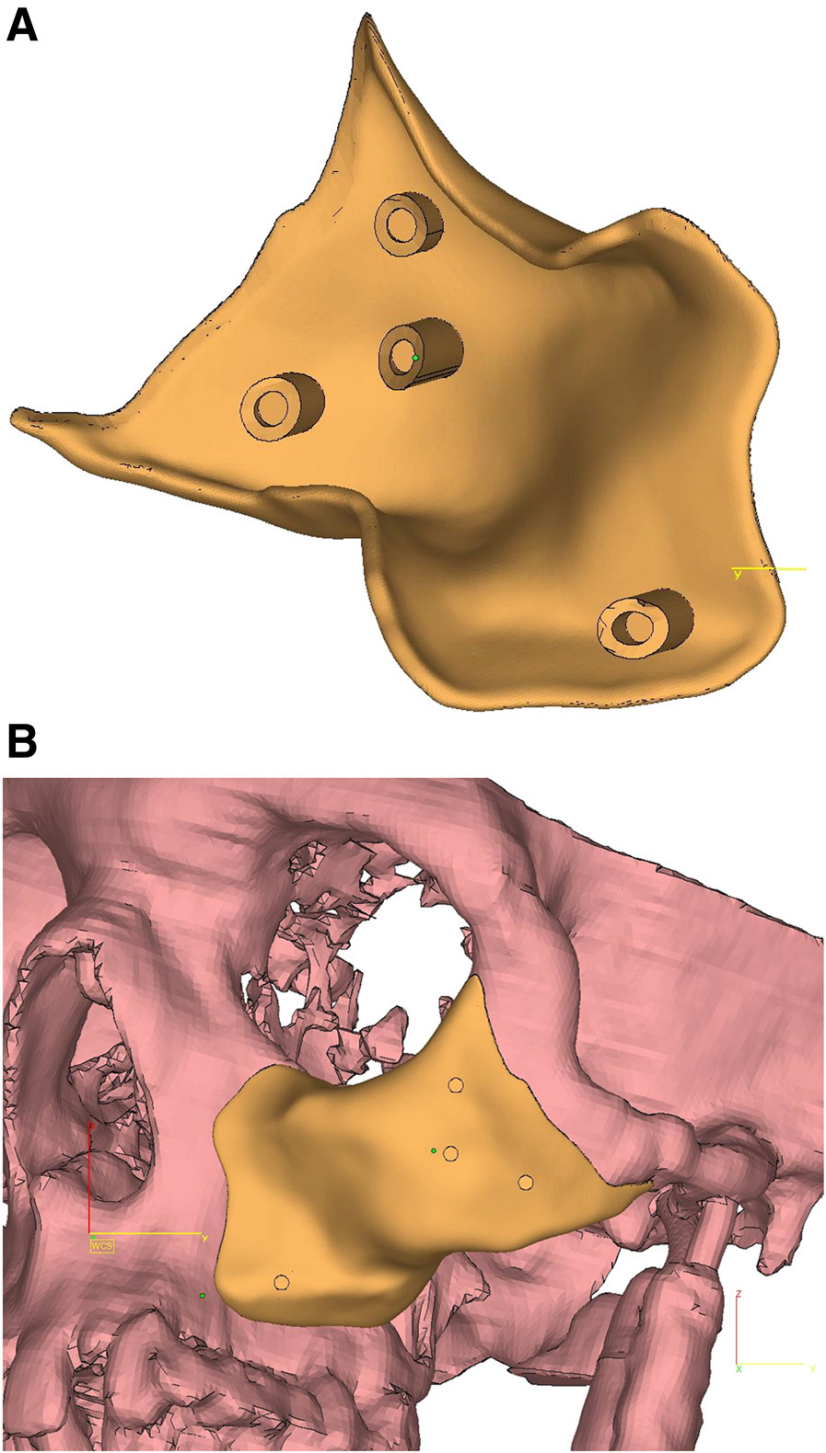
**The virtual zygoma implant. (A)** Internal side with fixation rods. **(B)** Position on the skull.

The zygomatic-orbito-maxillary defect was reconstructed using a custom-made titanium implant for esthetic reasons. Fine-cut CT scanning of the region with 3-dimensional (3D) reconstruction was performed (Siemens Somatom Sensation, Erlangen, Germany). The CT data were imported into the MIMICS® software (Materialise, Leuven, Belgium), and a 3D virtual model of the implant was produced by “mirroring” the healthy side using Freeform Modeling Plus® (3D Systems, Sensable, Valencia, CA, USA) platform software. Because a full-density titanium SLM implant would have been too heavy for implantation, we decided to produce an implant in the form of a shell that was supported by the residual bone and fixation rods (Figure 4).

The virtual model was then printed into the 3D implant by SLM using commercially pure titanium Grade 2 (SLM-Solutions, Luebeck, Germany) and an SLM 250HL machine (SLM-Solutions). The physical model of the skull was printed in white acrylic resin using Multi-Jet-Printing (Objet Eden 250, Stratasys, Eden Prairie, MN,USA). The SLM implant was placed on the plastic model of the skull to verify proper matching and seating. No further mechanical processing was needed. Finally, the produced implant was post-processed by sand-blasting and drilling the screw holes and then cleaned and sterilized by autoclaving.

**Figure 5:**
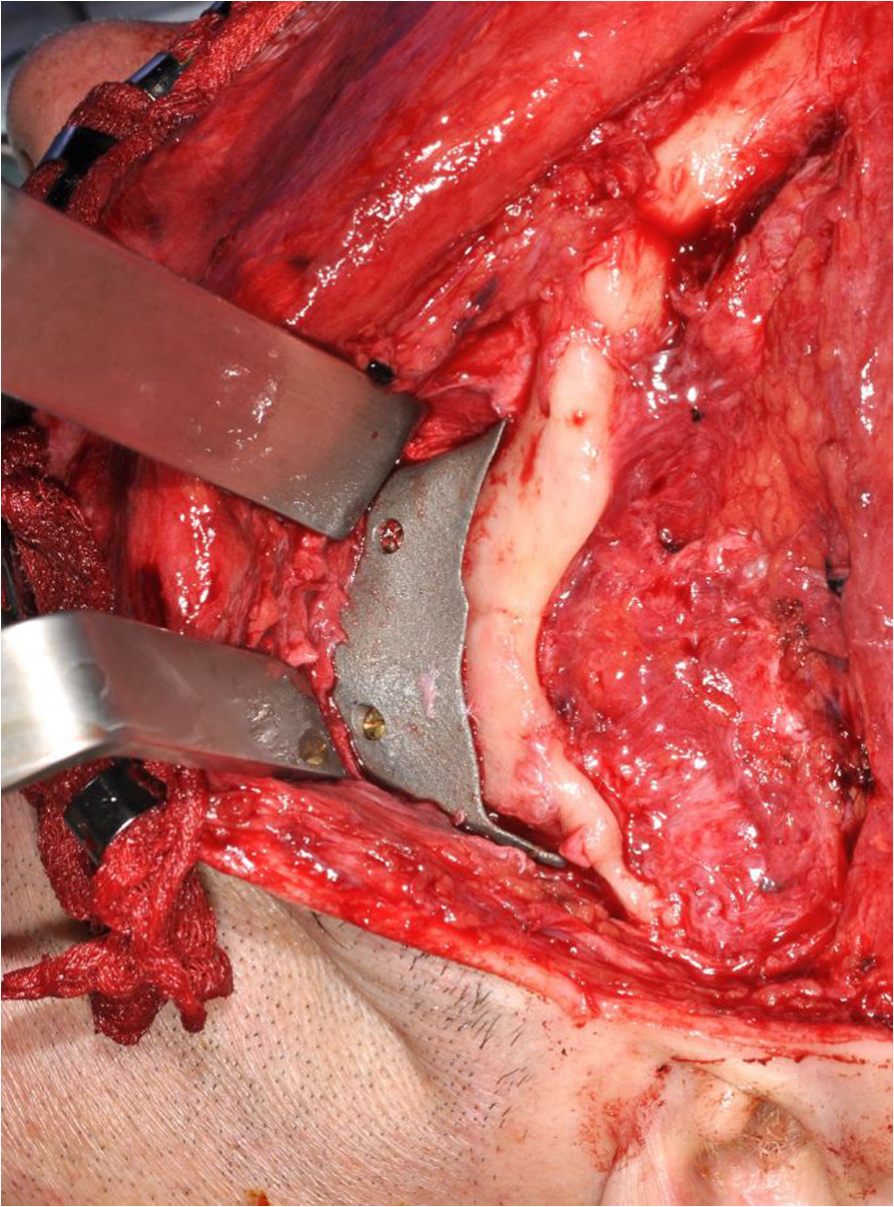
**Intraoperative placement and fixation of the implant using 2.0-mm titanium lag-screws(arrows).**

The implant was inserted into the planned position using a combination of mid-tarsal lower eyelid, hemicoronal and intraoral incisions. Proper seatings at the infraorbital rim, zygomatic body and zygomatico-alveolar buttress were confirmed. The fixation was performed with three 2.0-mm titanium screws (Stryker®, Michigan, MI, USA) using the lag-screw principle (Figure 5). The space between the shell-shaped implant and the residual zygomatic bone was filled with a cortico-cancellous iliac crest bone graft. The facial soft tissues were resuspended, and the left temporal hollowing was corrected with a titanium mesh. The wounds were sutured in layers and dressed appropriately.

**Figure 6:**
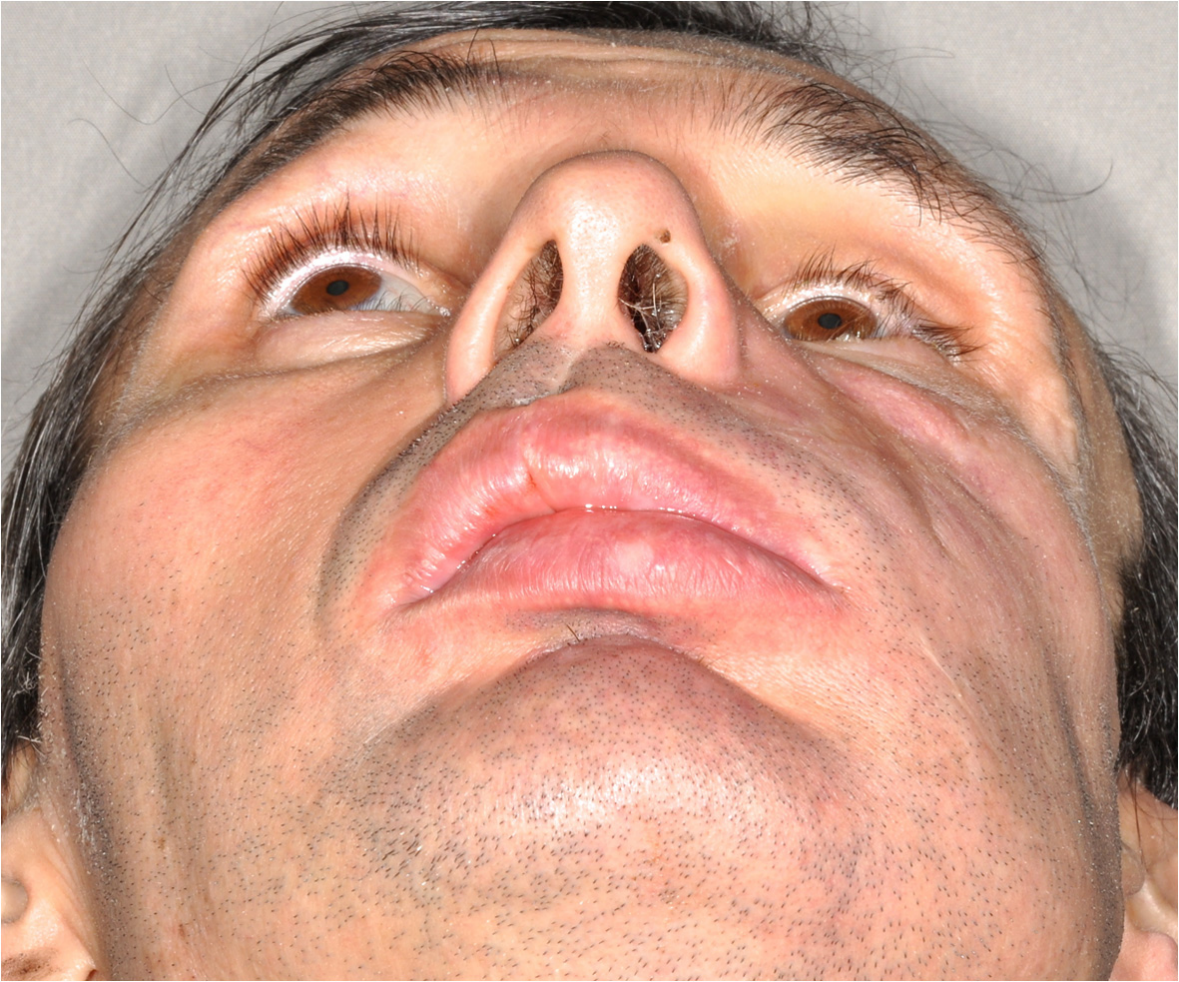
**Clinical appearance of the patient after the implantation.**

**Figure 7:**
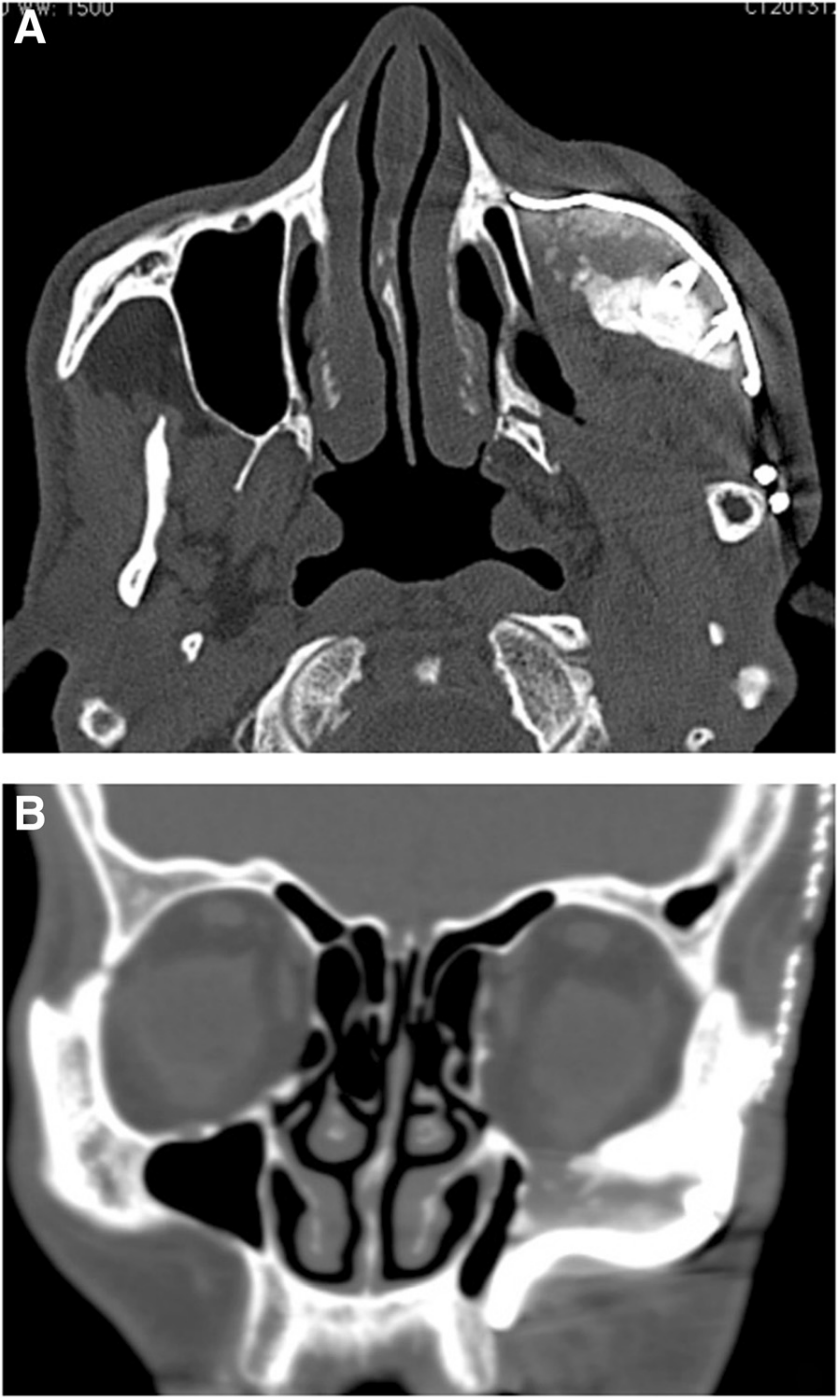
**Postoperative axial CT scan showing the restoration of the symmetry and anterior projection of the zygomatic bone: (A) Axial, (B) Coronal.**

The patient received 1 g of ceftriaxone, 80 mg of gentamycin, 0.5 g of metronidazole, and 100 mg of ketoprofen b.i.d. for 7 days. The postoperative course was uneventful, and the patient was discharged 8 days after the operation. The follow-ups at 1 month, 6 months and 1 year revealed no complications. At one year, the clinical examination revealed the persistence of a slight asymmetry in the zygomatic regions (Figure 6), and a CT scan supported the good projection of the reconstructed site and the symmetry between the two zygomas (Figure 7). We believe the residual asymmetry resulted from soft tissue atrophy. The CT scan also revealed good implant integration with ossification of the cortico-cancellous chips that were placed between the implant and the residual bone and no resorption of the residual zygoma.

## Discussion

Three-dimensional reconstruction of the zygomatic-orbito-maxillary complex is one of the most challenging procedures in craniofacial surgery. The normal anatomical contour and position of the zygomatic bone are critical for the appearance of the face [10]. Here, we described a successful use of a custom-made SLM titanium implant for the reconstruction of a post-traumatic zygomatic bone defect. Although the bony symmetry was maintained at one-year follow-up (Figure 7), the patient continued to exhibited soft tissue asymmetry. This asymmetry was most likely the result of buccal and zygomatic fat pad atrophy in response to the initial trauma and atrophy of the facial expression muscles secondary to facial nerve palsy.

Because the residual zygomatic bone was deficient in volume and shape, an osteotomy alone would have not properly corrected the projections. The patient rejected the option of free tissue transfer. Thus the only available option was alloplastic implantation. Different materials can be used in cases of post-traumatic zygomatic deficiency, the advantages and drawbacks of each of these materials have been thoroughly documented in the literature [6,8].

The development of CAD/CAM technology has opened new perspectives in the field of alloplastic implant production [11]. After three-dimensional reconstruction of a skull containing the defect, the future implant can be produced by “mirroring” the healthy side. Thus, the projection and symmetry of the zygomatico-maxillary complex can be re-established [7,11]. SLM is one of the CAD/CAM techniques that allows for the production of porous titanium parts that mimic bone structure [12]. Titanium is the most commonly used material in medical implants because it is highly biocompatible and integrates very well into tissues [13]. The mechanical properties of SLM titanium products are also within the ranges of the properties of bone [12]. These similarities are particularly important because implant materials that are much stiffer than the bone can generate stress shielding, which can potentially lead to bone resorption or hinder bone regeneration [14]. Bone resorption caused by stress shielding is believed to contribute to the aseptic loosening of implants [15]. In contrast, the porous surfaces of SLM titanium parts have been demonstrated to be favorable for cell adhesion, migration and ingrowth, and these properties result in strong bone-implant contact. When an implant is populated with osteogenic cells, these cells not only migrate on the surface of the implant but also inside the pores of the implant [13]. Due to advantages of the titanium structures produced by SLM, we designed and produced a patient-specific implant for zygoma recontouring using this technology (Figure 4). The implant fit perfectly into the defect, no corrections being needed at the time of surgery (Figure 5). Similar findings have been reported in the literature [7,9]. The implant was designed in the form of a shell and filled with cortico-cancellous chips from the anterior iliac crest to stimulate its integration. This implant behaved as reported in the literature [13], no complications or side-effects occurred.

The left eye enophthalmos was not corrected because anterior repositioning of the globe would have exposed a larger part of the cornea due to the presence of lagophthalmos. The limitation of the presented reconstructive procedure is that it addressed only the bony deficiency, leaving the soft tissue atrophy to be dealt with later. This atrophy could be corrected with structural fat grafting.

The SLM technique was an expensive procedure. However, the preoperative investment in time and technology was worthwhile due to the proper geometry of the implant, reduced operative time and the lack of donor site morbidity. These characteristics are consistent with those reported in the literature [7,9]. The drawback of the current study is the nature of single-patient case report, lacking sufficient follow-up. To reach definitive conclusions, extensive clinical studies should be conducted.

## Conclusions

In conclusion, custom-made alloplastic implants are particularly useful for zygoma recontouring making considerable contributions to the improvement of the final cosmetic and functional results. SLM titanium implants might be a promising alternative approach to alloplastic craniomaxillofacial bone reconstruction due to their geometrical, biological, and mechanical properties.

## Consent

Written informed consent was obtained from the patient for the publication of this report and any accompanying images.
