# Erratum to: Selective laser melted titanium implants: a new technique for the reconstruction of extensive zygomatic complex defects

**DOI:** 10.1186/s40902-015-0012-6

**Published:** 2015-04-08

**Authors:** Horatiu Rotaru, Ralf Schumacher, Seong-Gon Kim, Cristian Dinu

**Affiliations:** 1 Department of Oral and Cranio-Maxillofacial Surgery, “Iuliu Hatieganu” University of Medicine and Pharmacy, Str. Motilor Nr. 33, Cluj-Napoca, 400001 Romania; 2 School of Life Sciences, Institute for Medical and Analytical Technologies, University of Applied Sciences and Arts Northwestern Switzerland, Muttenz, Switzerland; 3 Department of Oral and Maxillofacial Surgery, Gangneung-Wonju National University, Gangneung, South Korea

## Erratum

After the “Competing interests” section in the original version of this article [1], an “Acknowledgements” section should be inserted and read as: “This study was partially funded by POSDRU grant no. 159/1.5/S/136893 - Parteneriat strategic pentru cresterea calitatii cercetarii stintifice din universitatile medicale prin acordarea de burse doctorale si postdoctorale - DocMed.Net_2.0".
